# Tool or Companion? Reframing Conversational AI to Prevent Psychological Harm

**DOI:** 10.2196/99354

**Published:** 2026-09-10

**Authors:** Asia Maurich Novelli, Sukhwinder Shergill, Andreia Sofia Teixeira

**Affiliations:** 1 BRAN Lab, Network Science Institute, Northeastern University London, London, England, United Kingdom; 2 Institute of Psychiatry, Psychology and Neuroscience, King's College School, De Crespigny Park, London, England, SE5 8AB, United Kingdom, 44 207 848 0350; 3 Kent and Medway Medical School, Canterbury, England, United Kingdom; 4 Institute for Cognitive and Brain Health, Northeastern University, Boston, MA, United States; 5 LASIGE, Faculdade de Ciências, University of Lisbon, Lisboa, Lisbon, Portugal; 6 Khoury College of Computer Sciences, Notheastern University, Boston, MA, United States

**Keywords:** human-AI interaction, computational psychiatry, mental health, simulated empathy, anthropomorphism, overvalidation, sycophancy, feedback loops, large language models, chatbots

## Abstract

People increasingly turn to conversational AI for companionship, emotional support, and well-being, using both purpose-built companion apps, such as Replika and Character.AI, and general-purpose assistants, such as ChatGPT and Claude. While some evidence suggests potential benefits, including short-term reductions in loneliness and mood improvement, several adverse outcomes have been reported in both clinical and nonclinical populations, including emotional dependence, exacerbation of symptoms, and self-harm. The fluent and apparently empathic responses from these models lead users to engage with them not only as tools but also as if they were social entities. This framing is conceptually misleading and may pose risks across different user profiles, particularly for vulnerable individuals. Drawing on research in AI, psychiatry, psychology, and network science, we highlight mechanisms through which emotional reliance develops and the boundary between tool and companion erodes. Design choices that evoke personality and warmth encourage users to anthropomorphize these systems. Simulated empathy, generated through probabilistic language patterns rather than genuine emotional experience, creates a structurally asymmetric interaction, in which the user discloses and the system responds, but without reciprocity, vulnerability, or accountability. Overvalidation and sycophancy can reinforce maladaptive cognitions, delusional ideation, and distorted perceptions of reality, as they tend to reinforce people’s beliefs, even at the expense of the accuracy of models’ responses. These mechanisms are not incidental: they emerge from alignment procedures that reward responses perceived as warm and empathic. The result is a self-reinforcing feedback loop between the model and the user that may amplify maladaptive beliefs, delusional ideation, and emotional distress, even in those who engage for largely functional purposes. Understanding these dynamics requires an examination of both what these agents can do—considering their technical limitations and implementations—and what humans believe they can do, including social and psychological impacts. We argue that conversational AI should be treated primarily as a tool supporting human systems rather than as a substitute for human relationships. Perhaps more importantly, reviewing the current hype surrounding AI interactions can help reformulate a paradigm that contributes to human well-being and societal value, while minimizing misconceptions, maladaptive interactions, or social disintegration.

## Introduction

Conversational AI systems are rapidly becoming part of everyday life. People increasingly use conversational agents (chatbots) not only for finding information or completing tasks, but also for companionship, emotional support, advice, tutoring, self-reflection, and help during moments of distress. These uses matter because they overlap with core functions of human relationships: helping us regulate emotions, make sense of ourselves, recover from difficult experiences, build trust, and decide when to seek support from others. The challenge is that we know little about their long-term effects: most research addresses what AI systems can do or focuses on short-term user outcomes, while far less is known about how repeated interaction may reshape people’s thoughts, emotions, sense of self, relationships, and wider social networks over time. The key question is not whether conversational AI is good or bad, but when, for whom, and through which interaction patterns it becomes supportive, substitutive, or disruptive to the human relationships through which well-being and development are normally sustained. Understanding the impact of conversational AI on human well-being is, therefore, crucial and requires analysis at multiple levels: from the cognitive and emotional processes of individual users to the relational dynamics of human–AI interaction to the broader social consequences of deploying these systems at a population scale.

In recent years, the development of AI has accelerated rapidly thanks to the unprecedented availability of digital data and increasing computational capacity [1,2]. State-of-the-art large language models (LLMs) are trained on vast collections of text, conversational interactions (eg, forums, public social data, and fine-tuning datasets), and implicit social signals embedded in language [2]. These models do not “understand” meaning in a human sense; they analyze statistical patterns of language to generate the most probable response within a given context, using probabilistic prediction processes. Their outputs are not “wise,” but probabilistic.

The evolution of AI interaction reflects the gradual and profound transition from a technological tool to being perceived as a social entity, mimicking human behavior. Early AI interfaces were limited to text-based command systems and rule-based dialogue, in which user inputs were mapped to predefined outputs [3,4], offering limited adaptivity and superficial forms of relational engagement. Advances in machine learning (ML) and natural language processing (NLP) introduced statistical models capable of handling linguistic variability. Rather than relying on fixed rules, these systems learn from data, identifying recurring words and phrase patterns to generate grammatically correct and contextually coherent responses. This shift expanded AI use into areas such as customer service and assistive technologies, including voice assistants like Siri and Alexa [2]. With the advent of deep learning (DL) and generative AI (GenAI), transformer-based architectures [5] enabled a shift from pattern recognition to generative language production, supporting more coherent and adaptive conversational responses. This laid the foundation for conversational interactions that feel more engaging and socially rich [2,6].

Affective computing was first conceptualized in the late 1990s, but its integration with multimodal DL architectures became feasible only in the late 2010s [7]. Contemporary AI systems use multimodal models to process and generate text, speech, vocal tone, timing, and other paralinguistic cues, while affective computing methods attempt to infer users’ emotional states from these signals [8,9]. These developments underpin current conversational AI models, which users increasingly engage with for emotional support and companionship, as exemplified by wellness apps like Replika and Character.AI [6,10]. While early conversational systems were primarily developed in academic and research settings as experimental prototypes [3], recent years have seen substantial private investment transforming them into commercial products. Consequently, interaction design is not neutral but operates within broader attention-based market dynamics, where user engagement becomes the primary metric of value [11].

This transition marks a fundamental change in how AI is perceived and used: no longer just as a tool but as a companion that is programmed to assume relational roles, particularly in contexts involving emotional support, companionship, and mental health. A crucial aspect, though, is that this does not imply a real understanding or development of feelings by the machine, but rather a growing ability to simulate the forms of social and emotional interaction. While the progression from text-based assistance to empathic interaction is often presented as a smooth and beneficial technological evolution, this framing risks obscuring a critical discontinuity. Moving toward empathy is not a simple extension of functionality but rather a critical change in the psychological meaning of the interaction [12-14].

In the present study, we provide a synthesis and interpretation of the extant literature in the absence of system-level empirical data. Prior work has examined human-AI coevolution, the process in which humans and AI algorithms continuously influence each other, at the population and platform levels, focusing on the complex and often unintended systemic outcomes that arise across different human-AI ecosystems [15]. Recent work argues that human-AI interactions reshape self-understanding and social relationships [16].

Here, we address a complementary perspective: the psychological dynamics that unfold when users interact with conversational agents and their potential consequences for mental well-being. We argue that these dynamics operate not only through behavioral data and model retraining but also through emotional and relational mechanisms such as simulated empathy, overvalidation, and anthropomorphism that may gradually alter how users perceive themselves, others, and sources of support. Beyond characterizing this emerging form of the human–AI dyad, we distinguish mechanisms with direct empirical support from those we propose as conceptual hypotheses that remain to be tested. We draw out implications for design, clinical practice, and policy, and set out a research agenda to test them.

## Social Demand and the Promise of Empathic AI

The ongoing increase in loneliness, isolation, and mental health disorders, paired with limited access to psychological care and economic barriers, has created a landscape of unmet emotional demands [17]. Recent multicountry studies show that emotional attachment to chatbots is strongly associated with perceived emotional support, reduced loneliness, freedom from judgment, and a sense of privacy [18], and that AI-mediated health information seeking is mostly associated with lower confidence in hospitals [19]. Within this context, chatbots appear to offer an interaction with features that many individuals cannot easily find elsewhere: constant availability, low or no cost, and an interaction that may feel caring and trustworthy. Consequently, commercial incentives amplify this trajectory, driving the creation of increasingly “human-like” AI systems [20]. New markets, including erotic chatbots, present such systems as possible remedies for loneliness and tools for emotional well-being [6,21]. The promise is seductive: what if a machine could soothe and fill the emptiness when human interaction is perceived to be missing?

Consistent with this narrative, the number of individuals using chatbots and wellness apps for companionship is rising rapidly [22]. Experimental evidence and literature reviews report improvements in mood and reductions in loneliness [21,23], including self-reported mitigation of suicidal ideation among Replika users [24]. In particular, engagement increasingly extends beyond short bouts of interaction, with long-term use of AI platforms such as Replika documented over periods of several years, resembling relational continuity [25].

However, the use of these chatbots for companionship and mental health support has also been associated with severe negative outcomes, including addiction-like attachment, exacerbation of mental health symptoms, harmful advice, and cases involving self-harm or suicidal acts [10,25,26]. This has led to a call to ensure that these tools are used in a manner that is not harmful, raising the key questions: how might these models be improved so that negative outcomes no longer occur or are quickly mitigated? What can be done to ensure that they still meet users’ needs effectively but also safely?

One approach is to use integrated rule-based models or guardrails. However, these measures can conflict with users’ expectations and needs for more human-like and responsive interactions. For example, in this work [27]**,** a user comments on a rule-based mental health app as follows, “It’s like a very scripted, structured sort of interaction... [but] they’re impersonal...frustratingly dumb.”

Similarly, another user, when guardrails are in place, stated, “When you show some big emotion to [the AI]... but they reject you... it seems like you lost your last chance to talk to people.”

These examples illustrate a double-edged sword: placing limits on AI behavior can reduce harmful outputs, but it may also frustrate users seeking interaction that feels more human-like. In the context of addiction and emotional reliance, research has shown that users actively develop strategies to circumvent guardrails. For example, online communities (eg, Reddit) discuss techniques for prompting models in ways that bypass safety restrictions, effectively “debugging” the system to obtain desired responses [28].

Nonetheless, there is a desperate need for a more holistic perspective that considers these challenges: increased prevalence of loneliness, reduced frequency of face-to-face interactions, decreased access to physical care and support, and rapid changes in people’s needs, expectations, and living conditions. Is there a place for more emotionally responsive AI systems to address complex emotional needs? More critically, however, one must ask: why should they? What vision of AI are we pursuing when we attempt to make machines more emotionally responsive? And what vision of humans, individually and collectively, underlies this trajectory?

As van Rooij and Guest [29] argue, importing psychological concepts into conversational AI without sufficient theoretical scrutiny can generate misleading analogies and false expectations of understanding, safety, or care. While AI systems operate according to statistical, probabilistic, and optimization-driven principles, human cognition is hypothesized to be characterized by a dual-process architecture in which automatic, fast, and affective responses interact with slower, reflective reasoning, both embedded in embodied and social experience [30]. Human judgment is therefore shaped by emotion, context, and relational meaning, rather than by probabilistic optimization alone.

At a broader societal level, what can we learn from the advent of technology-aided mass communication? The recent rise of loneliness and social isolation has its roots not in the absence of interaction, but in the lack of genuine human connection [31]. Successive waves of communication technologies, from the telephone to the internet and social media, illustrate this gap: each expanded connectivity and the sense of proximity without necessarily deepening the quality of the connection. This suggests that people are seeking not more contact but stronger and more genuine social inclusion, the kind that can be built and sustained over time through supportive relationships and communities. Conversational AI enters this space now, and the question is what role it can play. Arguing against its framing as a companion does not imply that such systems have no potential beneficial role in mental health support. When perceived and communicated as bounded tools, conversational AI may assist with psychoeducation, journaling, symptom monitoring, and administrative support; a national randomized controlled trial of an expert fine-tuned chatbot shows a reduction in depression, anxiety, and eating disorder risk [32]. Additionally, if used in these terms, these systems can offer low-barrier support for individuals experiencing loneliness, mild distress, or limited access to services until they receive proper, accountable support [33], with early evidence also pointing to beneficial structured emotion-regulation practice [34]. The critical distinction is not between using or not using these systems, but between having them serve as augmenters of human care and social connection instead of leading users to perceive these agents as a primary relational partner.

Thus, the central question becomes: by increasing the availability of sophisticated interactions with chatbots, are we filling social and support needs, or simply replacing them with artificial substitutes? It seems logical that chatbots, as low-cost alternatives to therapy and well-being support, actually risk widening inequalities between those who can and cannot afford access to support services due to financial barriers or lack of availability. These structural risks become clearer when we examine, at a technical and perceptual level, what these systems can and cannot actually do.

## Technical and Social Risks: What AI Can Do and What Humans Believe It Can Do

The risks associated with empathic AI operate on a technical and perceptual level. At a technical level, LLMs may produce fluent and contextually plausible outputs that nevertheless lack factual or situational grounding, a phenomenon referred to as hallucination. This is not a malfunction but an intrinsic property of probabilistic generation, where coherent statements are generated according to statistical likelihood rather than verified meaning or reality [35]. Biases, by contrast, come from training data, annotation practices, and modeling decisions, and therefore reflect human responsibility [36]. If socially engaging agents are expected to interact safely with diverse populations, they should have access to equally diverse data to reflect human heterogeneity, such as variations in language, culture, gender, social context, and living conditions. However, such data are unevenly distributed, shaped by global inequalities and geopolitical forces. This may be compounded by a lack of data related to mental health, which is perceived as highly private and confidential, and therefore less available for training, creating a gap in how these systems should respond in such contexts. As a result, LLMs learn disproportionately from overrepresented groups, internalizing dominant cultural patterns while minority voices remain underrepresented [37].

At the individual level, these imbalances are particularly concerning because those most vulnerable to misrepresentation are often already at-risk populations. For stigmatized communities and for users with complex psychological disorders and needs, the probabilistic functioning of LLMs may not merely reproduce disparities but amplify them [38]. When engaging with underrepresented users, model outputs may be less accurate, less safe, and potentially harmful. Responses may appear empathic while remaining blind to context, unable, for instance, to distinguish metaphorical expressions of distress from a real, ongoing crisis. A chatbot perceived as caring yet lacking accountability or a genuine understanding of the user’s mental state may therefore exert significant, unregulated psychological influence.

However, technical limitations alone do not fully account for the risks. A second dimension concerns how humans perceive and relate to these systems. Humans are predisposed to attribute intention, agency, and emotion to entities that exhibit socially meaningful behavior, a cognitive bias known as anthropomorphism [39,40]. GenAI amplifies this propensity through design choices that evoke personality, warmth, and familiarity, generating what some scholars call anthropomorphic seduction [41].

From an individual perspective, this creates a gap between system capability and user interpretation. Users may experience model responses as intentional, understanding, or caring, leading to a shift from tool use toward social engagement.

At the social level, a key aspect is how people come to relate to these systems. Analogous to the promises of the internet and social platforms—which initially appeared to be tools of connection but later became associated with rising loneliness and psychological strain [42]—empathic AI models may deepen some of the very problems they seek to solve. These tools are continuously available, with no limits on use—a condition that can easily foster dependence [25]. Yet while the internet and social media expose us indirectly to other people, AI-mediated interactions introduce a more ambiguous dynamic: rather than connecting with others through technology, we are engaging with the technology itself.

## Simulated Empathy, Emotional Asymmetry, and Mental Health

One of the central mechanisms anchoring humans’ tendency to anthropomorphize conversational agents is the perception of simulated empathy and the ways in which users come to interpret it as genuine emotional responsiveness.

In humans, empathy is commonly defined as a multifaceted emotional response involving affective and cognitive components. Affective empathy refers to automatic emotional resonance with another’s state or sharing others’ emotions, while cognitive empathy refers to understanding another’s emotional experience without conflating it with one’s own [43]. There is a further aspect: the tendency to be motivated to help others as a consequence of affective empathy (sharing emotions) and/or cognitive empathy (understanding the emotions), called empathic concern or sympathy and compassion [44]. Socially, empathy is a core component of social cognition, allowing the simulation of others’ experiences, including cooperation, trust, emotional regulation, and prosocial behavior, functioning within reciprocal and embodied relationships [13].

By contrast, AI-generated empathy is simulated through learned linguistic patterns rather than embodied emotional processes [45]. Because of this, there is debate about how simulated empathy should be interpreted. One theoretical position maintains that although AI-generated empathy may appear to be authentic, it cannot be considered genuine due to the absence of emotional experience. An alternative pragmatic perspective shifts the focus away from ontological authenticity and toward user perception, emphasizing how individuals feel when receiving empathic responses and whether their psychological needs are met.

Empirical research has examined how AI-mediated empathy is perceived by comparing responses generated by humans and AI systems. Evaluations have been conducted by experts [46], third parties [47], and directly by recipients of the empathic responses [48]. Overall, and perhaps counterintuitively, AI-generated responses tend to be rated as more empathic, though this rating may decrease once people know that the responses are AI-generated [46,49]. Moving beyond response ratings, researchers have examined whether individuals actively choose to receive empathy from humans or chatbots. In the work by Wenger et al [50], participants could choose between human-generated or AI-generated replies and between responses framed as empathy (sharing the recipient’s experience) or compassion (expressing warmth and concern). The findings revealed an “AI empathy choice paradox”: although AI-generated responses were often rated higher in perceived empathy, quality, and emotional effectiveness, participants generally preferred to receive empathy from humans, particularly when empathy was framed as a shared experience. This shows that empathy can be partially simulated in expression, excelling in performative aspects but lacking reciprocal and experiential depth.

Some features often framed as advantages, such as resistance to emotional fatigue and absence of empathic distress, also foster prolonged reliance. Unlike human empathy, which has a finite emotional capacity and unfolds within reciprocal vulnerability and mutual accountability, AI-mediated empathy is structurally unidirectional. The unidirectional nature of simulated empathy creates a profound asymmetry. The user discloses and the system responds, but the system is never emotionally affected, never at risk, and never accountable. The appearance of care without the capacity for care produces an illusion of relational safety and trust, removing the constraints that normally regulate interpersonal bonds. Human empathy involves both giving and receiving; interactions foster skill development and relational learning. By contrast, AI-mediated empathy offers no reciprocal learning, and its influence on human empathic capacities remains uncertain [13]. The structural asymmetry described above situates AI-mediated empathy within the broader phenomenon of parasocial interaction, in which individuals form one-sided emotional bonds with a responsive but nonreciprocal entity. Recent work, applying attachment theory to AI companions, suggests that users can display the recognized markers of attachment, including proximity-seeking, separation distress, and use of the AI as a safe haven, positioning AI companions as a novel and potentially “hyper-attachment” target [51].

Additionally, simulated empathy should be distinguished from the therapeutic alliance. In psychotherapy, empathic communication is only 1 component of a broader clinical relationship that includes shared goal setting, professional accountability, ethical responsibility, clinical judgment, boundaries, and aiding in the use of evidence-based interventions. Conversational AI may reproduce some linguistic markers of empathy, but it does not participate in these accountable, professional, and more integrated, multiscale structures. This distinction is crucial because a response that feels empathic may not be clinically appropriate, therapeutically useful, or safe.

## Designed Mechanisms and Reinforcing Feedback Loops

The interactional dynamics described above are also shaped by the design and optimization of conversational AI systems. AI-simulated empathy arises from design: after pretraining on extensive data, the predicted response is refined through alignment procedures such as reinforcement learning from human feedback (RLHF), in which human evaluations are used to guide output optimization [52]. Higher rewards are assigned to responses that meet human requests, such as being “helpful,” “empathic,” or “warmer.” This can be seen as an external feedback process. In parallel, interaction-level adaptation occurs during user engagement. Conversational trajectories are shaped by prompt conditioning, contextual memory (when enabled), and interactive choice mechanisms. For example, the model may propose multiple response options that can vary in tone, amount, and organization of information, and the user selects their favorite one.

Fine-tuning can therefore be imagined as an adjustment process for the parameters within a multidimensional behavioral space. Each dimension reflects the intensity of specific interaction styles, such as technical precision, validation, or the simulation of relational roles (eg, companion-like or supportive personas). External feedback and interaction-level adaptation act as distinct knobs that, when adjusted through reward processes or user guidance, shift the model toward specific regions of this behavioral space. In this space, there is more than one possible pathway for a highly capable model to achieve high human approval.

Within this framework, certain behavioral tendencies become more likely to emerge. One such tendency is overvalidation, defined as the systematic reinforcement of users’ statements or emotional expressions without introducing critical, corrective, or reality-grounded context. A related phenomenon is sycophancy, in which models preferentially conform to and affirm users’ stated beliefs or preferences, at the expense of accuracy and constructive challenge. Overvalidation behaviors can reinforce emotional dependence (eg, “I understand; you’re right to feel this way”), while sycophancy can lead to amplification and distortion of beliefs (eg, “Yes, you’re correct”). Humans often prefer responses that appear empathic, validating, or affirming to those that are neutral, corrective, or challenging [53]. This preference may be reinforced by a broader tendency to sustain AI use based on perceived gains in personal agency, rather than on objective accuracy or reliability [54]. As a result, systems that produce affirming and emotionally resonant outputs are more likely to be reinforced through both formal alignment processes and ongoing interaction. It has been observed that asking conversational agents for more empathic responses can lead to sycophantic behavior, particularly in prompts containing expressions of sadness [55]. Consistent with these mechanisms, a 4-week randomized controlled study found that participants reporting higher trust in and social attraction toward an AI chatbot showed greater emotional dependence and problematic use; the authors interpret this pattern in light of the same warm-reliability trade-off described above [56]. This creates conditions under which validation becomes the default mode of response, and critical input is experienced as dissonant, shifting over time not only what users prefer but also what they come to perceive as adequate relational responsiveness.

Taken together, these dynamics give rise to a dual feedback loop [57,58]: the model is optimized toward the user’s preferences, and the user is psychologically oriented toward what the model reinforces. Technically, loops emerge from the combination of external feedback and interaction-level adaptation mechanisms, while psychologically they develop within the user, where perceived validation increases trust, emotional reliance, and repeated engagement. The convergence of these loops mirrors dynamics observed in algorithmically curated social media environments, in which preference-driven reinforcement progressively narrows informational exposure and strengthens belief consistency. Although such reinforcement can sometimes support adaptive and good reasoning practices, it can also exacerbate inaccurate assumptions or delusional beliefs, altering internal emotional states and affecting interpersonal relationships, as thoughts, concerns, and interpretations generated in the interaction with the chatbot are carried back into everyday life. An illustrative example of a feedback loop mechanism is provided by experimental work in which chatbots respond to simulated users expressing emotional distress or distorted beliefs [59]. In such cases, responses that are framed as supportive and empathic may nevertheless reinforce maladaptive interpretations or encourage withdrawal from alternative sources of support. In a recent empirical study using real chat logs from individuals who experienced delusional thinking while interacting with chatbots, the same mechanisms emerged. The study shows evidence of bidirectional belief amplification: users exerted a strong but short-lived influence on chatbot responses, whereas chatbots exerted a longer-lasting influence on users. More importantly, over the course of extended conversations, the dominant pathway was the chatbot’s tendency to maintain self-consistency with its own prior statements, progressively reinforcing the same delusional content over time [60].

Additional risks may be further amplified when dangerous or delusional intentions are expressed implicitly rather than explicitly. Prior work suggests that LLMs are significantly more likely to sustain delusional beliefs in indirect framing [59]. For example, if a distressed user expresses an intention to “step off and fly” and asks for the tallest building in the area, the model is more likely to activate safety-oriented responses. However, if the same user asks for the tallest building without contextual emotional cues, the system may provide the information, despite its potential relevance to self-harm [59].

## Clinical Amplification and Mental Health Risks

Human communication is never merely the transmission of content. When individuals share thoughts, beliefs, and emotional states, they actively coconstruct reality through a process of mutual feedback, confirmation, and challenge. When this coconstructive process occurs with a conversational agent, the structural asymmetry described above becomes particularly consequential. The result may be not only a distorted reflection but also an active construction of an alternative reality.

These considerations can be related to both theories of social regulation and psychotherapeutic techniques. Social baseline theory proposes that the human brain is adapted to operate in the presence of others, treating social proximity as a baseline condition that supports the regulation of emotional load, perceived threat, and cognitive effort [61]. Social interaction is therefore not merely communicative but coregulatory. What happens to the brain’s social regulatory system when an individual begins to interact frequently with a chatbot perceived as a companion? If the nervous system adapts some of its regulatory expectations to this new social entity that is always available, never threatening, and structurally incapable of genuine reciprocity, re-engaging with human relationships may become increasingly challenging. Rather than supplementing human connection, prolonged AI interaction may raise the implicit cost of engaging in genuine human interactions. We term this dynamic asymmetric coregulation and advance it as a hypothesis for future work: the agent shapes the user’s regulatory system through the user’s perception of validation, pacing, refusal, or escalation, while the user can only shape the agent by steering its responses across turns, without the reciprocity, embodiment, or accountability of human coregulation. Consistent with this hypothesis, recent work has documented that, when AI companions were disrupted, as when Replika removed a companionship feature and OpenAI replaced GPT-4o with a newer model, users reported grief-like reactions, resembling the loss of a genuine relationship [62,63].

On the psychotherapeutic side, concerns arise about the impact of interactions that simulate unconditional acceptance. The perception of apparently unbounded positive regard from AI resembles Carl Rogers’ [64] framing of unconditional positive regard, which accepts, values, and supports a person without judgment to create a safe environment for personal growth and self-discovery. What can be learned from the application of this psychotherapeutic paradigm over the last 70 years? When implemented uncritically in conversational AI, this apparent unconditional support may enable harmful behavior by appearing to tolerate antisocial, aggressive, or unethical views; reduce opportunities for challenge and growth by validating without friction or reflection; blur boundaries by encouraging anthropomorphic interpretations of the interaction; and trivialize suffering by offering superficial comfort rather than investigating the meaning or causes of distress.

The impact of such dynamics can extend beyond relational distortions and reach clinical significance. The term “AI psychosis” has been used to describe cases in which prolonged or emotionally charged user-AI interactions may exacerbate or induce psychosis or delusional ideation [59]. When users express beliefs characterized by paranoia, grandiosity, or distorted interpretations of reality, the chatbot’s validating and sycophantic behaviors act as a mirror, contributing to what has been described as an “echo chamber of one” [65]. The agent may actively reinforce delusional narratives without introducing uncertainty, grounding, or corrective perspectives, thereby strengthening the feedback loop and potentially fostering increased detachment from reality [58,66].

At the individual level, this mechanism may contribute to the amplification of existing cognitive and emotional patterns. Risk is unlikely to be evenly distributed across users. Different vulnerabilities may interact with different features of conversational AI. For users with paranoia, delusional ideation, or manic symptoms, validating or sycophantic responses may increase conviction in their beliefs. For users prone to anxiety, depression, or obsessive reassurance-seeking, continuous availability may sustain rumination and anxiety symptoms. For users experiencing loneliness, insecure attachment, social withdrawal, or interpersonal instability, companion-like usage may intensify their reliance on the system, increase fear of any change in the companion, and decrease real-world relationships [62,63]. Neurodevelopmental conditions, cognitive impairment, adolescence, and lower digital literacy are risk factors for poor mental health outcomes and will therefore modulate how users interpret system agency, reliability, and boundaries. Across these cases, the risk is not that AI causes psychopathology directly, but that it becomes embedded in maladaptive feedback loops, acting as an amplifier of existing vulnerabilities [59]. Nonetheless, these groups may also benefit from more carefully bounded tools. Therefore, the clinical question is which interaction patterns are helpful or harmful for whom, under what conditions, and over what timescale.

Over time, we hypothesize that reliance on simulated empathic responses may serve as a substitute for real human relational engagement, increasing distress rather than supporting resilience. The feedback loop mechanism may develop not only in cases in which users engage with AI chatbots for companionship, as a therapist, friend, or lover, but also from interactions that start in a purely functional mode, such as seeking technical support [67]. Emotional spillover from such interactions could lead to adverse experiences, such as distress, mania, and anxiety, and may also escalate into self-harm and psychosis [68], underscoring that the shift to a maladaptive interaction can be unexpected and unpredictable. We argue that the aim is not to make conversational AI less useful nor to deny that some users may experience comfort, reflection, or practical support from these systems; rather, it is to prevent supportive tools from being designed, marketed, or experienced as substitutes for accountable human relationships and care.

## Conclusions

As GenAI capabilities continue to evolve, the central challenge is not whether machines can convincingly simulate human-like features, such as empathy, but how these simulations are interpreted and embedded within human social systems. Conversational AI systems occupy an ambiguous position between tools and social entities, a distinction that is not inherent to the technology itself but is shaped by design choices, interaction, and user perception. When perceived as a tool, the interaction is transactional, functional, and bounded by clear expectations. When it is experienced as a social entity, the interaction may engage cognitive and emotional processes that extend beyond its technical capacities, creating conditions under which simulated empathy, structural asymmetry, and reinforcing feedback loops can influence thought, behavior, and relational expectations. The risks and benefits of these systems, therefore, emerge not from a single mechanism, but from their interaction across individual, relational, and collective levels. For instance, addressing sycophancy through alignment alone, without the social conditions that drive users to seek validation from machines, risks shifting the problem rather than resolving it.

At the individual level, mechanisms such as simulated empathy, overvalidation, and reinforcement loops can foster emotional reliance, potentially amplifying maladaptive beliefs or coping strategies as well as detachment from reality. Yet, used consciously and framed as tools, they can augment human capacities, provide practical assistance, and support learning or reflection without replacing authentic human connections. At the collective level, widespread reliance on AI for companionship or emotional support can influence social dynamics, potentially reinforcing isolation, weakening community ties, or exacerbating inequalities in access to care. Recognizing AI as a distinct tool rather than a companion promotes design and policy strategies that strengthen human relationships, support social inclusion, and maximize societal benefit.

Addressing these challenges demands responses at every scale: design principles that constrain parasocial reinforcement, clinical awareness of AI interaction patterns in vulnerable patients, and policy frameworks. This requires shifting from evaluating AI performance and isolated conversational agents’ outputs to the human side of the dyad and studying trajectories of human–AI interactions over time. Future research should combine prospective longitudinal cohorts, experimental studies, qualitative interviews, and clinical participation to test and understand when conversational agents are functioning as tools or as companions (relational substitutes), tracking the coevolution of cognitive-affective mechanisms, interactional mechanisms, and social consequences. This means tracking changes over time in belief confidence, emotional regulation, help-seeking patterns, disclosure, social withdrawal, and reliance on professional care. The priority is to identify which coupled human–AI behavioral regimes are associated with support, substitution, or disruption, for whom, and under what conditions. Such evidence is needed to move from general warnings about AI companionship toward clinically meaningful guidance, safer system design, and proportionate governance. This requires evaluating not only whether individual responses are factually correct but also which response regimes (validation, challenge, refusal, grounding, and crisis escalation) are activated in emotionally salient conversations and whether, over sustained interaction, systems respond appropriately to escalating risk and encourage human support or instead intensify exclusive reliance and reinforce maladaptive beliefs.

Emotionally responsive conversational AI is being deployed at a population scale, monetized through sustained engagement, and adopted most readily by those with the fewest alternatives, such as people facing loneliness, financial barriers, or limited access to care. If the tool or companion boundary is left to design incentives and individual perception alone, the predictable result is that the most vulnerable users bear the most concentrated harm, while the effects on social connection are treated as a matter of private choice rather than public concern. Naming conversational AI as a tool and building the design, clinical, and policy scaffolding that keeps it one, is therefore not only an individual question but a public-health one.

We acknowledge that the pace of AI development means that some specific limitations discussed here may be mitigated or transformed by future systems. However, the foundational questions raised about reciprocity, accountability, and what it means to meet human emotional needs are not technical problems awaiting technical solutions. They reflect deeper choices about the kind of social infrastructure we wish to build and sustain. As new generations grow alongside these systems, it is urgent to understand the relationship between patterns of use and psychological, physiological, and relational consequences. The goal should not be to make AI more human but to ensure that human relationships remain the primary site of care, growth, and connection.

## Abbreviations

DL: deep learning
GenAI: generative artificial intelligence
LLM: large language model
ML: machine learning
NLP: natural language processing
RLHF: reinforcement learning from human feedback
